# Mutational Analysis of Early, Low-Grade Bowel Polyps Defines a Subgroup with Concurrent, High-Risk Oncogenic Drivers Independent of Polyp Size

**DOI:** 10.1158/2767-9764.CRC-25-0182

**Published:** 2025-08-19

**Authors:** Jun Li, Zoe Welham, Benita Tse, Chahaya Gauci, Shila Ghazanfar, Pratibha Panwar, Alexander Engel, Mark P. Molloy

**Affiliations:** 1 Bowel Cancer and Biomarker Laboratory, Kolling Institute, School of Medical Sciences, The University of Sydney, Sydney, Australia.; 2 Colorectal Surgical Unit, Royal North Shore Hospital, St. Leonards, Australia.; 3 Sydney Medical School, The University of Sydney, Sydney, Australia.; 4 School of Mathematics and Statistics, The University of Sydney, Sydney, Australia.

## Abstract

The accumulation of pathogenic mutations in premalignant colorectal neoplasms is critical for colorectal cancer development; however, the frequency and diversity of pathogenic driver mutations in early-stage colorectal polyps is incompletely known. We investigated this by whole-exome sequencing of low-grade dysplasia (LGD) colorectal polyps and paired germline DNA. Interestingly, in these early lesions, there was no association with mutational burden and the known risk factor of polyp size (range, 3–15 mm). However, a subset of LGD polyps harbored concurrent, oncogenic alterations in colorectal cancer–causing pathways of WNT/β-catenin, p53, or RTK-RAS. Further analysis suggested that the concurrent mutation signature was associated with increased polyp burden. Spatial transcriptomic analysis revealed that immune effectors, including NF-κB signaling, were a characteristic of LGD polyps with concurrent pathogenic mutations. Together, these observations suggest that some small-sized, early colorectal neoplasms have enhanced oncogenic potential and highlight that nonadvanced colorectal adenoma may not be universally considered a low-risk finding.

**Significance:**

Through whole-exome sequencing of early colorectal polyps characterized by LGD, we demonstrate that polyp size poorly correlates with mutational burden. Some small-sized (<10 mm polyps) early polyps harbored concurrent driver gene mutations commonly seen in adenocarcinoma. Sequencing of key driver genes in early polyps may identify risky lesions that cannot be detected by histology alone.

## Introduction

Colorectal cancer maintains a high worldwide disease burden as the third most common malignancy and the second leading cause of cancer mortality (1). Colonoscopy with subsequent excision of bowel polyps has been proven as an effective intervention to reduce colorectal cancer incidence and mortality (2, 3). Based on clinical guidelines, risk assessment for metachronous adenomas and neoplasia after removal of colonic polyps may depend on the size, histology, and number of polyps detected and removed (4). High-risk factors are polyp size of ≥10 mm that confers a 3% annual risk for colorectal cancer development, villous architecture that confers a risk of 17%, or high-grade dysplasia that already has a risk of 37% (5–7). Despite the well-known benefits of screening, the accuracy of pathologic risk factors for informing the timing of surveillance colonoscopy is debated (8), leading to variability in guidelines according to jurisdictions (4).

Adenomas and carcinomas are infrequent outcomes of a pervasive process of neoplastic change across morphologically normal colorectal epithelium developed through a continuum acquisition of oncogenic mutations (9). At least 23% of somatic mutations are already present in at-risk mucosa prior to adenoma initiation, and deregulation of the WNT pathway is required to foster carcinogenesis (10). Recently an ultrasensitive, single-molecule mutational test based on CRISPR technology revealed that the presence of oncogenic *KRAS* and *TP53* mutations in normal colon mucosa is a common event in patients with colorectal cancer (11). These results emphasize that cancer risk, especially established oncogenic drivers of initiation and progression, might exist amongst the molecular changes at the earliest stage of clonal evolution of colorectal cancer and provide motivation to consider molecular characteristics as additional colorectal cancer risk factors which could inform screening.

A few studies (12–14) have explored the possibility that the mutational information of bowel polyps, comprising a small panel of genes, might help to predict the risk of metachronous advanced neoplasia. Other biomarkers like tumor mutation burden (MB) have also been investigated in colorectal cancer and polyps. Sequencing studies have demonstrated the trend of increasing MB along the order of low-grade dysplasia (LGD), high-grade dysplasia, conventional colorectal adenomas, non-hypermutated colorectal cancer, and hypermutated colorectal cancer (10, 15). A previous study used CT colonography to measure the growth rate of small polyps and found that conventional adenomas with two to three pathogenic mutations had a mean growth rate of 60% compared with those harboring a single pathogenic mutation, which had a mean growth rate of only 13%. This observation suggests there is a relationship between pathogenic mutation load and polyp development (16).

Nonetheless, small-sized (<10 mm) and LGD colorectal polyps are commonly considered low-risk lesions for colorectal cancer. However, the substantial rate of interval cancers (17, 18), many developing from small, missed lesions, suggests that some early-stage colorectal polyps harbor inherent oncogenic features that belie a low-risk classification. In this study, we sort to better characterize the breadth of oncogenic drivers found in early stages of colorectal carcinogenesis. We focused on bowel polyps with LGD and conducted whole-exome sequencing (WES) from polyp biopsies and paired germline DNA to identify the somatic mutation landscape. Spatial transcript profiling was used to investigate gene expression in a subgroup of mutationally distinct LGD adenomas. Together, the study highlights a subset of LGD bowel polyps with oncogenic features suggestive of enhanced risk.

## Materials and Methods

### Ethical approval

The study was conducted in accordance with the Declaration of Helsinki, and all participants provided written informed consent. The study was approved by the Northern Sydney Local Health District Human Research Ethics Committee (2019/ETH00301).

### Patient and public involvement

Two consumer representatives with bowel cancer lived experience were involved in the design of this study.

### Endoscopy unit description

The study center is a quaternary referral hospital and was undertaken in conjunction with the Department of Colorectal Surgery during the period 2020 to 2023. All colonoscopies were performed by, or under the direct supervision of, an experienced endoscopist certified in colonoscopy by the Conjoint Committee for the Recognition of Training in Gastrointestinal Endoscopy which sets rigorous minimum standards in Australia and whose accreditation can only be awarded to medical practitioners with specialist registration. Endoscopists must maintain, through compulsory and centralized audit, at least 95% caecal or terminal ileum intubation rates, adenoma detection rates of >25%, and a >4% sessile serrated adenoma/polyp detection rate.

### Study design

This study used the Strengthening the Reporting of Observational Studies in Epidemiology cohort reporting guidelines. All patients, excluding those with a history of familial polyp syndromes, Lynch syndrome, or inflammatory bowel disease, scheduled for colonoscopy were asked to participate in the study. If colorectal polyps were found at colonoscopy, a 2 mm^3^ polyp biopsy, using standard colonoscopic biopsy forceps, from a maximum of two different polyps was taken and immediately snap-frozen. Polyp biopsy strategy was unselected, that is, the first polyp encountered on the way in was biopsied, and if present, a second polyp immediately proximal to the first polyp was biopsied. Snare polypectomy was performed, and the specimen was removed, placed in formalin, examined by a certified practicing subspecialized pathologist, and reported synoptically according to the Royal College of Pathologists of Australasia (19). Reported polyp size, after biopsy, was taken from the definitive pathology report. In line with standard clinical practice, diminutive rectal hyperplastic polyps (HP) were not routinely removed. Blood was collected into EDTA tubes and used for genomic DNA extraction.

### Patient data

A total of 272 patients undergoing scheduled colonoscopy consented to participate in the study; of those, 177 patients had no neoplasia detected. When a neoplasm was detected, it was biopsied for research. A total of 95 neoplasms were biopsied, and following DNA and sequencing quality control (QC) assessment, 79 specimens from 62 patients returned useful WES data (Supplementary Fig. S1; Supplementary Table S1).

Study size was informed by available grant funding and timeline. Indications for colonoscopy included symptomatic patients with per-rectal bleeding, fecal occult blood test positive screening, those with a family history of colorectal polyps or colorectal cancer, and those under scheduled colonoscopic surveillance with a personal history of colorectal polyps or colorectal cancer. Table 1 shows the clinical characteristics of participants.

**Table 1:** Study cohort and histology summary

Number of participants	62
Number of specimens	79
Number of polyps	70
Gender	​
Female	20 (32.3%)
Male	42 (67.7%)
Age (years)	​
Range	28–88
Mean (SD)	63.03 (13.90)
BMI (range)	​
Female	26.7 (16–43)
Male	25.9 (13–35)
Neoplasia type	​
Hyperplastic	10 (12.7%)
TA	44 (55.7%)
TVA	9 (11.4%)
SSL LGD	7 (8.9%)
Adenocarcinoma	5 (6.3%)
Normal mucosa	4 (5.1%)
Polyp size	​
Small <10 mm	51 (72.9%)
Large ≥ 10 mm	19 (27.1%)
Polyp site	​
Proximal colon	35 (50.0%)
Distal colon	35 (50.0%)
Indication of colonoscopy	​
Personal history polyps	14
Rectal bleeding	14
FOBT+/FIT+	8
Personal history colorectal cancer	7
First-degree relative colorectal cancer history	3
Other^a^	16

a Other includes abnormal gastrointestinal imaging scan and patient-reported change in bowel habit exceeding 14 days

Abbreviations: BMI, body mass index; FIT, fecal immunochemical test; FOBT, fecal occult blood test.

### WES

In total, 79 bowel biopsies and paired germline DNA extracted from blood underwent Illumina WES using SureSelect Human All Exon V7 capture kit (Agilent Technologies). DNA extraction from biopsies and blood followed the manufacturer’s protocol of QIAamp Fast DNA Tissue Kit and QIAamp DNA Blood Midi Kit (QIAGEN), respectively. Polyp and paired germline DNA targeted 200× and 100× reading depths, respectively. The bioinformatic pipeline followed the steps of genomic alignment using BWA-MEM, marking duplicates by Picard (RRID: SCR_006525), base quality score recalibration, and realignment around local insertions–deletions (indel) by GATK4.0 (20). Somatic variant calling was done by Strelka2 (21) and Manta (22). Annotation and gene summary statistics were generated by VEP (23) and maftools (24). Oncogenic variants were determined according to the annotation of OncoKB_Annotator (25). Microsatellite instability (MSI) status in polyp specimens was estimated by MSIsensor-pro (26).

### Spatial transcriptomics

To examine the impact on gene expression resulting from concurrent mutations, we subjected nine formalin-fixed, paraffin-embedded polyp samples consisting of three with concurrent mutations and six with nonconcurrent mutations (Supplementary Table S2) to spatial profiling using the GeoMx Digital Spatial Profiler (DSP; RRID: SCR_021660) Cancer Transcriptome Atlas (CTA) panel (NanoString). Five-micrometer-thick sections were prepared and processed following GeoMx DSP manual slide preparation workflow (MAN-10150-04). After antigen retrieval, tissue sections were hybridized with CTA probes and then stained using morphology markers pan-cytokeratin (PanCK), CD45, and SYTO13 for cell nuclei. In each region of interest, the segmentation was conducted using the following rules: PanCK+/CD45^−^ for epithelial cells and CD45+/PanCK− as immune cells. The captured probe library (8,659 probes) was sequenced on the NextSeq 1000 system for P2 100 cycles paired-end sequencing for read 1, i7 and read 2, i5 sequences, respectively.

### Spatial transcriptomic data analysis

The FASTQ files were generated in the BaseSpace Sequence Hub (RRID: SCR_011881) and downloaded to the local server for input to the GeoMx NGS tool pipeline (RRID: SCR_023424) following GeoMx NGS user manual (NanoString, MAN-10153-06). The Digital Count Conversion files were uploaded onto the GeoMx DSP for exploration with the Data Analysis Suite (NanoString). The dataset contained expression of 8,659 probes in 145 segments obtained from polyp and normal regions of nine tissue samples (Supplementary Table S2). The GeoMx DSP Data Analysis Suite was used to perform data QC, which included (i) a segment QC step that assesses and flags technical noise level (e.g., low negative probe count) and other issues at the sample level (e.g., low nuclei count) and (ii) a bioprobe QC step to identify outlier probes and generate gene-level expression. Due to technical issues, one of the segments did not contain any reads and was removed from the dataset. Following this, the expression data for 1,812 genes (including one negative probe) in 144 segments was downloaded and analyzed with R packages.

For differential gene expression analysis, segments with nuclei count less than 50 were filtered out, leaving a total of 139 segments for this analysis. To compare gene expression differences between segment groups, the generalized linear model functions glmFit() and glmLRT() in the R package edgeR v4.4.1 (27) were used on raw gene expression counts (excluding negative control). *P* values were corrected with the Benjamini–Hochberg method using the topTags() function in the edgeR package (RRID: SCR_012802). For the comparison between concurrent and nonconcurrent groups, we focused on concurrent polyp epithelial segments versus nonconcurrent polyp epithelial segments (PanCK+) and concurrent polyp immune segments versus nonconcurrent polyp immune segments (CD45^+^). An adjusted *P* value of 0.05 (−log_10_*P* value of 1.3; GLM likelihood ratio test; Benjamini–Hochberg method FDR <0.05) and log_2_ fold change (FC) of 0.5 were used as cutoffs.

### Immune cell deconvolution analysis

To assess the relative abundances of immune cells in the 144 segments, the R package Consensus^TME^ v0.0.1.9 (28) was used. Applying the consensusTMEAnalysis() function from the package to the Q3 normalized gene expression counts (excluding negative control), with colon adenocarcinoma (COAD) as reference cancer and single-sample gene set enrichment analysis as the statistical method, generated the enrichment scores for 18 cell types provided in the Consensus^TME^ package. Statistical significance of differences in cell type enrichment scores from polyp versus normal immune segments and concurrent versus nonconcurrent polyp immune segments was tested following a two-sample *t* test approach with t.test() in the base R stats package. To account for FDRs, the *P* values were adjusted using the Benjamini–Hochberg correction with p.adjust() in the base R stats package.

### Statistical analyses

Correlation between MB and polyp size was tested by the Kendall rank correlation test in R v4.1.2. The Wilcoxon rank-sum test was conducted in R v4.1.2 with the package *rstatix* v0.7.0 to compare the MB between large- and small-sized polyp groups.

### Data availability

WES data are available from the NIH BioProjects repository with the identifier PRJNA1040952. The GeoMx raw data used in this article have been uploaded to Zenodo (https://doi.org/10.5281/zenodo.14847288). Other data generated in this study are available upon reasonable request from the corresponding author.

## Results

### Polyp histology and whole-exome MB

Of 272 consented participants, 177 had no detectable neoplasia, whereas 95 patients had suspected neoplasia biopsied and subsequent polypectomy was performed (Supplementary Fig. S1). The mean age of this cohort was 63 (range, 28–88). Of the participants, 67.7% were males (Table 1). Thirty-three patients were excluded as the biopsy specimen DNA quality was too poor for sequencing. Whole exomes were obtained from 79 biopsies from 62 participants. Histopathology review of sequenced biopsies determined these were obtained from 53 conventional adenomas, 10 HPs, 7 low-grade sessile serrated lesions (SSL), 5 conventional adenocarcinomas, and 4 normal mucosa. All polyps were reported as LGD. Fifty-one polyps were small (<10 mm), which consisted of 36 tubular adenomas (TA), 2 tubulovillous adenomas (TVA), 10 HPs, and 3 SSLs; the remainder were large (≥10 mm). Of 51 small polyps, 17 were classified as diminutive (<5 mm; Supplementary Table S1).

**Figure 1:**
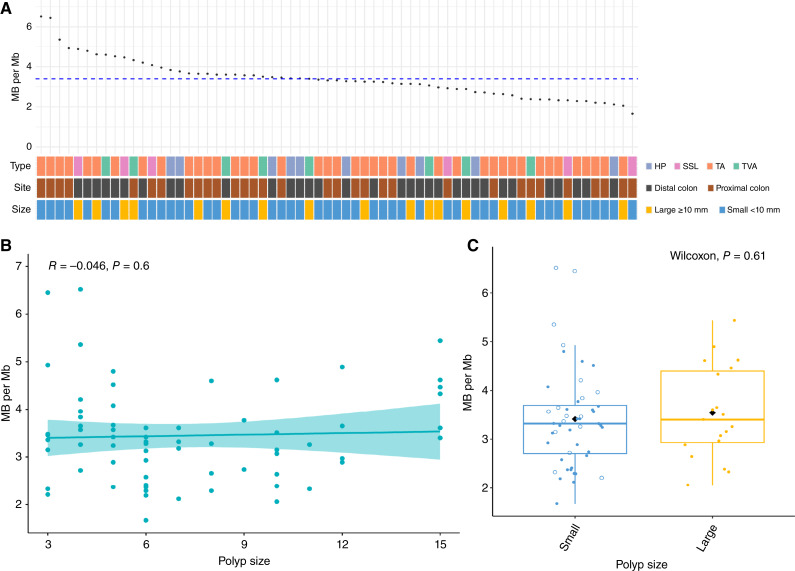
MB in colorectal polyps (*n* = 65). **A,** Distribution of MB and polyp type, location, and size (mean MB–blue dashed line). **B,** Kendall rank correlation test between MB and polyp size (shaded area shows the 95% confidence interval). **C,** Comparison between polyp MB and pathology-reported polyp size (mean MB–black diamond; the top whisker is the maximum value plus 1.5 × IQR, and the bottom whisker is the minimum value minus 1.5 × IQR). Open circles are diminutive polyps (<5 mm).

The WES capture kit targeted >99% of human exons, and after removing PCR duplicates, 81.07% to 97.99% reads had greater than 20× coverage of target bases, with a mean coverage 139× (91× to 205×) across all specimens. The average MB of polyp specimens was 3.39 (1.67–6.52) mutations/Mb sequenced (Fig. 1A), whereas five microsatellite-stable (MSS) adenocarcinomas had an average MB of 4.45 (3.3–5.32). Considering polyp type, the mean MB was 3.24 (2.12–3.84) in HPs, 3.41 (1.67–4.89) in low-grade SSLs, 3.39 (1.77–6.52) in TAs, and 3.52 (2.39–4.62) in TVAs. None of the biopsies were MSI-high when assessed using the MSIsensor-pro bioinformatic tool (Supplementary Fig. S2). We observed that one small TA (257plb) with the highest MB of 6.52 possessed a predicted deleterious missense variant p.T278K (SIFT score = 0) in the POLE DNA polymerase exonuclease domain, which is associated with hypermutated tumors (29), providing an example in which mutational data would indicate additional disease risk that could not be determined by histology alone.

### Mutation spectrum

The top 10 mutated genes for each histologic polyp subtype (TA, TVA, HP, ad SSL) are reported in Supplementary Fig. S3. To assess the mutational profile of bowel polyps, we excluded WES biopsies of five histology-confirmed polyps as they did not show any oncogenic variants, having a molecular profile consistent with normal colon mucosa, suggesting that biopsies were taken from healthy mucosa at the polyp edge. After also separating the adenocarcinomas, this produced a final polyp cohort of 65 from 52 patients for subsequent analysis.

### Conventional adenomas

**Figure 2:**
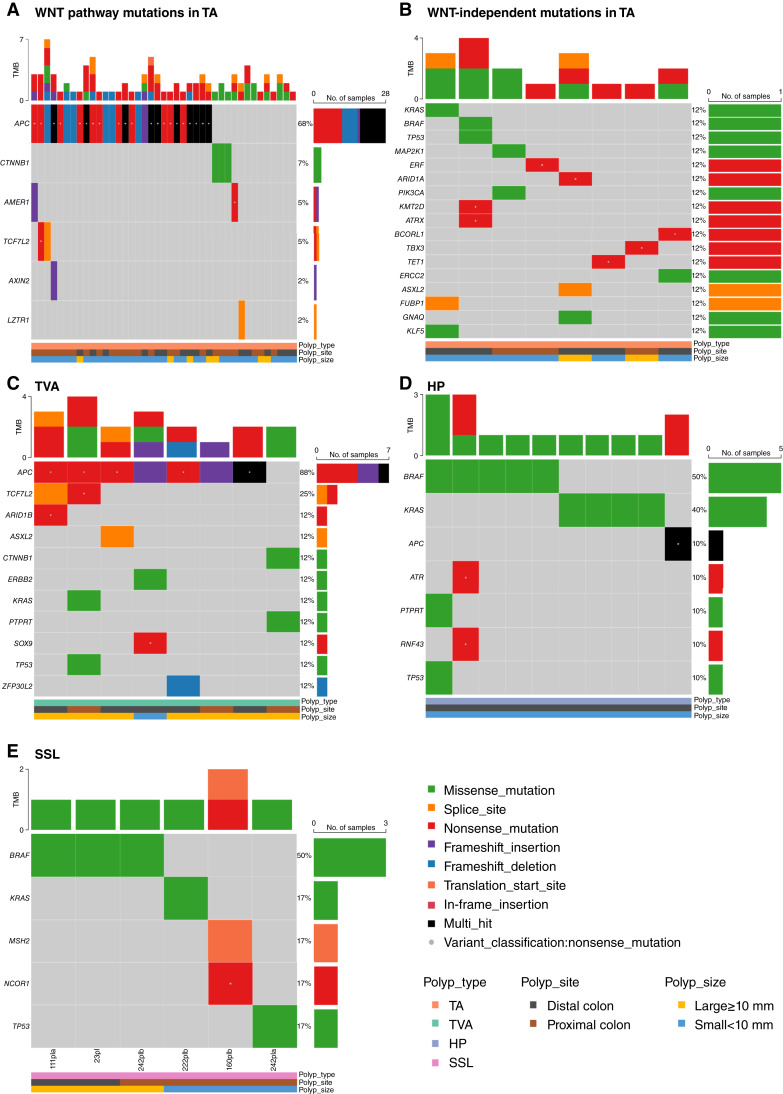
Oncoplots of bowel polyps. **A,** WNT pathway alterations in TAs. **B,** Oncogenic mutations of eight WNT-independent TAs. **C,** Oncogenic mutations in eight TVAs. **D,** Oncogenic mutations in HPs. **E,** Oncogenic mutations in SSLs. TMB, tumor MB.

In 41 TAs, some of the most frequently mutated genes were *APC* (71%), *DNAH2* (24%), *RYR2* (17%), *DNAH5* (17%), *LRP2* (17%), *MADCAM1* (15%), *MYO15A* (17%), *APOB* (15%), *ROS1* (15%), *CTNNB1* (12%), and *EP400* (12%; Supplementary Fig. S4). In total, ∼80% TA polyps carried pathogenic mutations in WNT pathway genes, of which 29 TA polyps exhibited 43 exonic variants in *APC*, with the four remaining TA polyps characterized by *CTNNB1* activation mutations [recurrent p.T41A (rs121913412) and p.S45F (rs121913409)] and a stop-gain mutation *AMER1* p.R601* (Fig. 2A)*.* The exonic *APC* variants found in TAs are comprised of 8 frameshift indels, 29 nonsense, 4 missense, and 2 splice-site variants. In the TA cohort, ∼20% had non-WNT pathway mutations comprised of four polyps with OncoKB listed oncogenic mutations in the RTK-RAS pathway (*KRAS* p.D33E, *BRAF* p.V600E, *MAP2K1* p.K57N, and *ERF* p.E202*), and 4 other TAs had oncogenic mutations in *ARID1A* p.R1276*, *ASXL2* p.X135_splice, *GNAQ* p.T96S, *TET1* p.R745*, *TBX3* p.E179*, *BCORL1* p.R1420*, and *ERCC2* p.R631H (Fig. 2B). The polyp with *ERF* p.E202* also had a nonsense variant *NRAS* p.R102* with uncertain pathogenicity (Supplementary Table S3). TET1 (30) is a tumor-suppressor that inhibits colon cancer growth by derepressing inhibitors of the WNT pathway, and TBX3 (31) acts as tissue-specific component of the Wnt/β-catenin transcriptional complex. As the TAs represent the largest number of specimens obtained, we investigated for differences in gene frequency between large and small TA polyps; however, no significant differences were detected.

Of eight TVAs, all eight showed *APC* and one had *CTNNB1* as driver oncogenic mutations (Fig. 2C). Two of eight carrying *APC* mutations also have *KRAS* p.G12V/D, which is a general oncogenic feature that was also observed in the five MSS colorectal adenocarcinoma samples also sequenced in our study (Supplementary Fig. S4).

### HPs and SSLs

*BRAF* p.V600E (50%) or *KRAS* (p.G12V/D, 40%) mutations were present in 90% of HPs, with one other HP carrying *APC* p.R213* (rs587781392) which has been reported in the ClinVar database to be associated with familial adenomatous polyposis and *APC*-associated cancers (32). Interestingly, one small distal HP had co-occurring oncogenic mutations in *TP53* p.P151S (rs28934874) and *PTPRT* p.R1343Q (rs775489787) in addition to *BRAF* p.V600E (Fig. 2D), whereas the second distal lesion carried nonsense mutations in *ATR*, *RNF43*, and *BRAF*.

In six SSLs reported with LGD, four possess *BRAF* p.V600E or *KRAS* p.G12V mutations. The remaining two SSLs are <10 mm and have oncogenic driver mutations in *TP53*, *MSH2*, and *NCOR1* (Fig. 2E).

### Concurrent oncogenic mutations in LGD polyps

We downloaded somatic variants reported from normal healthy colon from SomaMutDB (33) and The Cancer Genome Atlas (TCGA) COAD/READ tumor mutation data to investigate differences in oncogenic pathways between normal colon tissue, colorectal adenocarcinomas, and variants identified from bowel polyps in this study. Broadly, the bowel polyp samples reflected variants reported in TCGA adenocarcinoma data, with WNT, RTK-RAS, TP53, NOTCH, and Hippo as the most frequently altered pathways in LGD bowel polyp specimens examined in this study (Supplementary Fig. S5). Normal colon tissue showed minimal mutational alterations in those pathways. Examining the TCGA COAD/READ dataset, 77% of colorectal cancer has concurrent mutations in either two of three pathways: Wnt/b-catenin, RTK-RAS, or TP53 (Supplementary Fig. S6).

**Figure 3:**
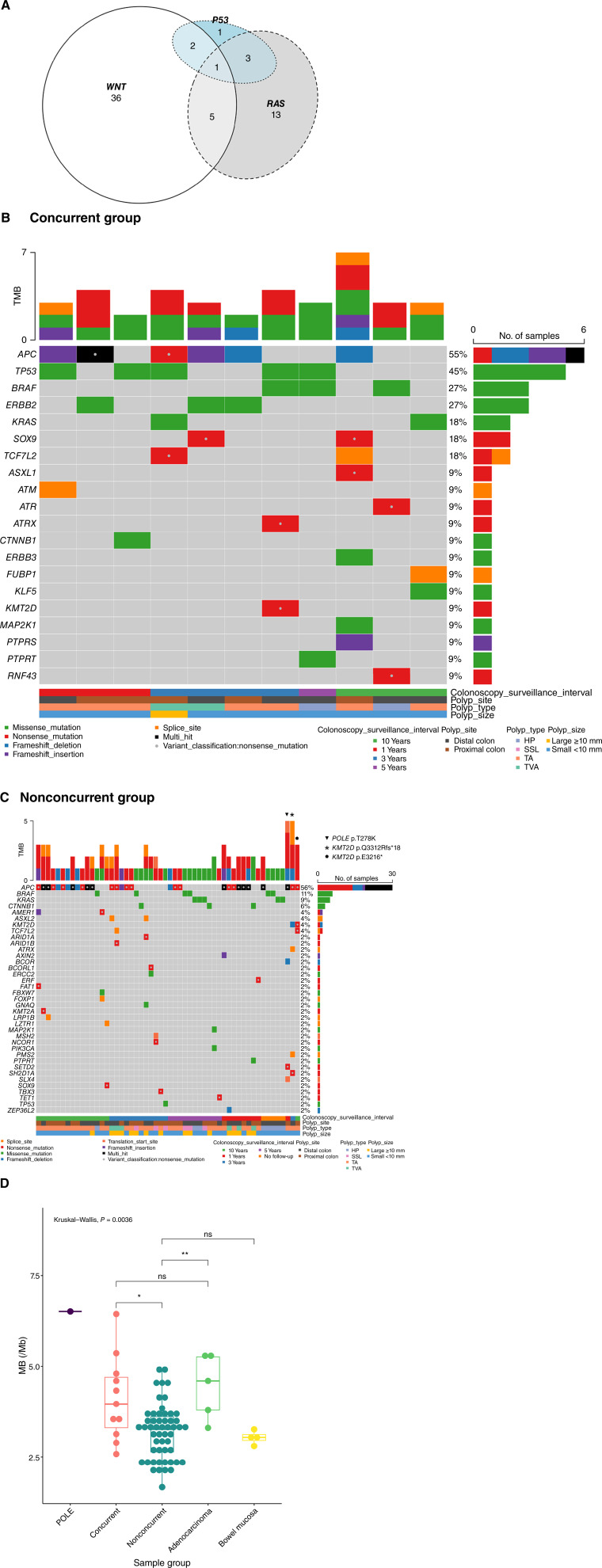
Oncogenic mutations defining a concurrent mutation signature in bowel polyps. **A,** Venn diagram to show the selection criterion of polyps with concurrent mutations in two of three molecular pathways. **B,** Oncogenic mutations in low-grade polyps with concurrent mutations. **C,** Oncogenic mutations in low-grade polyps without concurrent mutations. **D,** Box plot of MB between polyp groups, colorectal cancer CRC, and normal mucosa (top and bottom quartiles and mean are shown; the top whisker is the maximum value plus 1.5 × IQR, and the bottom whisker is the minimum value minus 1.5 × IQR). TMB, tumor MB; ns, not significant.

Focusing on the most commonly altered colorectal cancer driver pathways, we observed a subset of polyps with concurrent oncogenic/likely oncogenic mutations according to OncoKB (25) that affect high-risk carcinogenesis pathways of Wnt/b-catenin, RTK-RAS, or p53 (34). The frequency of these variants in NOTCH and Hippo pathways was minor and absent, respectively. This was observed in 11/65 LGD colorectal polyps, and 10/11 LGD polyps were <10 mm in size (Fig. 3A and B; Supplementary Table S4). Additionally, we highlight three other polyps with oncogenic mutations in other known cancer-causing pathways. Two polyps with defects in WNT signaling also showed *KMT2D* mutations p.E3216* and p.Q3312Rfs*18 which have been reported in tumors with high tumor MB that often respond favorably to immunotherapy (35). One small TA polyp (257plb, 4 mm) had *POLE* exonuclease domain variant (p.T278K) in addition to multiple hits in *APC* (p.R1114*, p.E1544*), a frameshift indel in *BCOR* (p.V1065Sfs*48), and a nonsense mutation in *SETD2* (p.S1045*). The remaining 51 LGD polyps (Fig. 3C; Supplementary Table S5) showed oncogenic mutations affecting only a single colorectal cancer driver pathway, consistent with early-stage tumorigenesis.

Those polyps with concurrent mutations in Wnt/b-catenin, RTK-RAS, or p53 had a significantly higher mean MB of 4.11 compared with those without these concurrent mutations (MB = 3.19). Of the five MSS adenocarcinomas we profiled, the mean MB was 4.45, whereas the four normal bowel mucosa specimens had a mean MB of 3.03 (Fig. 3D). We examined possible confounders. Age greater than 50 years showed a weak positive correlation with MB (*R* = 0.21), whereas body mass index was not correlated with MB (Supplementary Fig. S7).

### Relationship between polyp size, polyp burden, and MB

Previous investigation has shown that MB increases from adenomas to MSS colorectal cancer, to MSI-high colorectal cancer (10). Polyp size and grade are used as a cutoff threshold to define advanced adenoma. Given our observation of distribution of MB in LGD polyps, we compared MB with polyp size, which surprisingly revealed no correlation (*R* = −0.046, *P* = 0.6). This poor relationship of size and MB was consistent within each polyp subtype and between different polyp subtypes (Fig. 1B; Supplementary Fig. S8; Supplementary Fig. S9). MB was not significantly different using 10 mm to define polyp size, which is commonly considered a risk stratification cut point (Fig. 1C). Interestingly, we observed high MB for some individual cases of diminutive conventional adenomas. For example, a 3-mm TA harbored one of the highest MB of 6.45, with pathogenic mutational defects in numerous colorectal cancer driver genes (e.g., *APC*, *SOX9*, *TCF7L2*, *ASXL1*, *ERBB3*, *MAP2K1*, and *PTPRS*, Supplementary Table S6).

We investigated the correlation between polyp burden and MB in concurrent and nonconcurrent groups (Supplementary Fig. S10). For polyps with concurrent mutations, we observed a significant positive correlation between polyp burden and MB, whereas no association was observed in the nonconcurrent mutation group, highlighting the likelihood for increased polyp burden to be associated with the concurrent mutation signature.

### Gene expression profiling in polyps with concurrent oncogenic mutations

**Figure 4:**
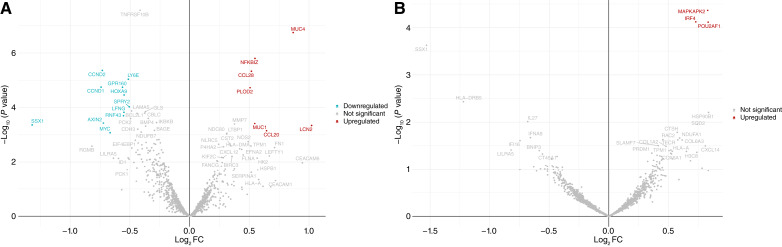
Volcano plots showing differentially expressed genes between different polyp histologic segments. **A,** Epithelial segments (PanCK+) of polyps with concurrent mutations epithelial segments vs. polyps without concurrent mutations. **B,** Immune segments (CD45^+^) of polyps with concurrent mutations vs. polyps without concurrent mutations. An adjusted *P* value of 0.05 (−log_10_*P* value of 1.3; GLM likelihood ratio test; Benjamini–Hochberg method FDR <0.05) and log_2_ FC of 0.5 were used as cutoffs. Red denotes genes increased in relative expression, blue denotes genes decreased in relative expression, and gray denotes genes that did not exceed differential expression thresholds.

To explore how gene expression may be affected in bowel polyps with concurrent oncogenic mutations, we subjected a subset of nine formalin-fixed, paraffin-embedded adenomas (three concurrent and six nonconcurrent mutations) to spatial profiling using the GeoMx CTA panel. Comparing the gene expression in PanCK+ epithelial regions of polyps containing concurrent driver mutations with those with single driver mutations revealed 7 upregulated transcripts (log_2_ FC > 0.5) and 11 downregulated transcripts (log_2_ FC < −0.5). Included within the upregulated genes in the concurrent mutation group were some previously linked with colorectal cancer carcinogenesis, including *NFKBIZ*, *CCL20*, *LCN2*, *PLOD2*, *MUC1*, and *MUC4* (Fig. 4A; Supplementary Table S7). When comparing immune segments (CD45^+^) in polyps with concurrent mutations against nonconcurrent specimens, *MAPKAPK2* and *POU2AF1* were significantly upregulated genes (Fig. 4B).

We attempted to identify the relative abundances of immune cell types based on ConsensusTME (28) immune cell deconvolution analysis; however, there was heterogeneity that prevented identification of significant differences between sample groups. Some trends were noted, suggesting an immune-suppressed microenvironment in polyps such as fewer macrophages, dendritic cells, and CD8 T cells. We saw the same trend of fewer CD8 T cells as well as reduced NK cells, and increased T regulatory cells in polyps with concurrent mutations compared with those with single driver mutations (Supplementary Fig. S11; Supplementary Table S8).

## Discussion

This study characterized the mutational landscape of early colorectal polyps with LGD. A key finding was that approximately 15% of small-sized, LGD polyps carried concurrent pathogenic driver mutations in key colorectal cancer signaling pathways, WNT, RTK-RAS, and p53, like those commonly seen in colorectal cancer. That is, for every 1,000 small polyps encountered, we can expect that approximately 150 will have similar concurrent mutations in key colorectal cancer driver pathways.

In contrast to colorectal cancer, only limited studies have characterized the molecular relevance of sporadic gene mutations in premalignant bowel neoplasia. Lin and colleagues (12) characterized the mutational alterations using WES and proposed four genes (*CTNNB1*, *KRTAP4-5*, *GOLGA8B*, and *TMPRSS13*) as the potential somatic drivers in conventional adenomas. We only saw *TMPRSS13* (a silent rs1198073770) in one 5-mm TA and *KRTAP4-5* (rs238830 missense) in one 15-mm large TVA, but *GOLGA8B* was not detected in our cohort. These genes are absent from OncoKB Cancer Gene List (1,164 genes, last update October 24, 2024) and not reported to involve in colorectal cancer driver pathways. Sievers and colleagues (16) investigated the mutation profile in early colorectal polyps by targeted sequencing of 50 cancer driver genes and correlated this with measurements of polyp growth rate. Conventional adenomas with two to three pathogenic mutations were more likely to be growing than those with a single pathogenic mutation. We extended from this study by utilizing germline-paired WES to obtain highly accurate somatic information from whole exonic regions of LGD colorectal polyps. We made the important observation of a subset of early polyps with concurrent mutations in WNT, RTK-RAS, or p53 signaling pathways, and these lesions had higher mean MB than polyps lacking concurrent mutational defects in these colorectal cancer driver pathways (Fig. 3D). Understanding the association between concurrent oncogenic mutations in these three signaling pathways and incidence of metachronous neoplasia is now critical to advance how this information could be utilized for risk stratification strategies.

Amongst the polyps with concurrent mutations, there are some interesting observations to highlight. Two cases were small distal HP lesions, of size 3 and 6 mm, which are mostly considered as benign lesions. The 3-mm polyp carried oncogenic mutations *TP53* p.P151S (rs28934874), *BRAF* p.V600E (rs113488022), and likely pathogenic mutation *PTPRT* p.R1343Q (rs775489787). The 6-mm HP had *BRAF* p.V600E (rs113488022) and nonsense mutations *ATR* p.R2193* and *RNF43* p.R371* (rs771831816; Fig. 2D). RNF43, a cell surface transmembrane E3 ubiquitin ligase, regulates WNT signaling by promoting the ubiquitination and lysosomal degradation of FZD (36). Truncating mutations of *RNF43* are more prevalent in MSI tumors and show mutual exclusivity with inactivating *APC* mutations in colorectal adenocarcinomas (37). Two other cases arose from small TAs. A 5-mm rectal TA harbored *KRAS* p.D33E, *FUBP1* p.X115_splice, and *KLF5* p.P301R. *FUBP1* has been identified as a long tail cancer driver and globally affects the landscape of alternative splicing to create aberrant proteins that drive malignant transformation (38). Previous studies have suggested that *KLF5* has an important role in cancer cell proliferation, and recurrent missense mutations in the phosphodegron domain of *KLF5* have been identified in colorectal carcinomas (39). Finally, we observed a case with two 3-mm TA polyps in the proximal colon; both showed highly oncogenic mutational disruptions affecting *APC*, *KMT2D*, *TCF7L2*, *SOX9*, *ASXL1*, *ERBB3*, *MAP2K1*, and *PRPRS*, consistent with advanced tumorigenic capacity. From a risk stratification perspective, diagnosis of these small polyps assigned the participants to long surveillance intervals of 5 to 10 years. Whereas these polyps were excised, it is tempting to speculate that the mutational profiles are protumorigenic and may be well suited to transformation given more time. It is therefore reasonable to propose that small, missed lesions with similar advanced mutational spectrum could develop as an interval cancer between colonoscopy periods. This is relevant as estimates suggest as many as 43% to 52% of colorectal interval cancers are because of missed lesions (17, 18). Current guidelines for colonoscopy surveillance timing rely on histopathology diagnosis to measure polyp villosity, high-grade dysplasia, and/or polyp size ≥10 mm as risk factors to define advanced adenoma. Whereas these factors have histologic utility, our data show that they only coarsely reflect the underlying genetic alterations which are the key drivers of malignancy.

Spatial transcriptomic profiling found that the expression of *NFKBIZ*, *PLOD2, MUC1*, and *MUC4* were elevated in PanCK+ epithelial segments of polyps with concurrent mutations, whereas *POU2AF1* was observed in the immune compartments of those polyps. *NFKBIZ* encoding IκBζ is a key gene in the regulation of a variety of inflammatory factors in the NF-κB signaling pathway (40). Unlike cytoplasmic IκB proteins, IκBζ is localized to the nucleus in which it regulates NF-κB–specific gene expression. Increased IκBζ level is associated with activation of proinflammatory mediators, including IL-17c, IL-19, and LCN2, and CCL20 (41), which we also observed. CCL20, a critically important chemokine, belongs to the subfamily of small cytokine CC. In colorectal cancer, CCL20 has been shown to be dysregulated, with elevated levels observed in tumor tissues and associations with cancer progression and poor clinical outcomes (42). One of the key mechanisms by which CCL20 exerts its effects is by activating the NF-κB signaling pathway (43). Stimulation of NF-κB signaling in colorectal cancer has been linked to tumor growth, resistance to apoptosis, enhanced metastatic potential (44), and decreased survival.

PLOD2 has been reported as an oncogenic gene in colorectal cancer and functions by stabilizing USP15, leading to AKT/mTOR-regulated progrowth signaling. Overexpression of PLOD2 has been shown to facilitate colorectal cancer proliferation, invasion, and metastasis, both *in vitro* and *in vivo* (45). Expression of the mucin transcripts MUC1 and MUC4 was higher in polyps with concurrent mutations. As well as protecting the epithelial layer from bacterial attack and mediating adhesive properties, these genes can modulate important cancer signaling pathways. For example, MUC4 interacts with ERBB2 to induce phosphorylation and can also enhance expression of CDKN1B to promote G1 cell-cycle growth (46, 47).

Elevated expression of *POU2AF1* transcripts was observed in the immune segment. POU2AF1 is a transcriptional coactivator for POU2F1/OCT1 and POU2F2/OCT2. It specifically recognizes the POU domains in these target genes to promote their transcription activity (48), which includes cytokine induction upon macrophage activation (49) and CD4 T-cell (50) differentiation. POU2F2/OCT2 expression is highly specific to the B-cell lineage, induced by NF-κB pathways (51). Interestingly, IκBζ functions as a coactivator of both POU and NF-κB transcription factors. IκBζ can amplify and integrate the output of NF-κB and POU transcription factors at inducible genes in immune cells (52). We note the limitation of this analysis was the inclusion of one high-grade TA (*APC* and *TP53)* in the concurrent oncogenic mutation group*,* which was due to the limited availability of specimens suitable for GeoMx*.* Despite this limitation, the above analysis highlights that neoplasms with concurrent mutations in important colorectal cancer driver pathways also regulate gene expression of some immune effectors, potentially enhancing the carcinogenic capacity of these lesions.

A strength of this study was its prospective design, with colonoscopy performed under the supervision of a single experienced clinician at a single institution. We focused on exonic gene mutations, whereas other factors, including epigenetics and copy-number variations (CNV), are variables known to influence cancer development and were not considered in this study. Whereas CNV analysis can be reliably performed from whole-genome sequencing data, detecting CNVs from WES data is error-prone due to technical difficulties, including uneven coverage during targeted enrichment, GC content, and sequencing biases (53, 54). To date, CNV callers using WES data suffer from low precision and concordance, meaning that extensive validation is required to substantiate findings (55). The current study provides an interesting hypothesis for further exploring risk in early bowel neoplasia which will include any associations of the concurrent oncogenic mutations with metachronous neoplasia.

In summary, this study highlighted the diversity of oncogenic mutations associated with early colorectal neoplasia from low-grade lesions. We note that the linear sequence (56) of gene driver mutations proposed in the adenoma–carcinoma sequence (i.e., *APC* followed by *KRAS* followed by *TP53*) was not strictly observed in our study. This is explainable due to the advancement in sequencing technologies to uncover gene drivers that were not detectable in earlier periods and highlights the importance of three driver pathways of WNT, RAS, and p53 in promoting growth of early colorectal polyps.

## Supplementary Material

Supplementary Figure S1 Study recruitment and analysis workflow.

Supplementary Figure S2 MSI scores in the sample cohort, estimated by MSIsensor-pro.

Supplementary Figure S3 Top ten mutated genes in each bowel neoplasia type.

Supplementary Figure S4 Oncoplots of bowel polyps and five adenocarcinoma specimens.

Supplementary Figure S5 The most frequently altered oncogenic pathways reported in TCGA adenocarcinoma data was mapped to bowel polyp specimens analyzed in this study (tubulovillous adenoma (TVA), tubular adenomas (TA), sessile serrated lesions (SSL) and hyperplastic (HP), and normal healthy colon data.

Supplementary Figure S6 Venn diagram to show the intersection between CRC carrying mutations in WNT, RAS and TP53 pathway from TCGA adenocarcinoma dataset. "total" indicates the whole sample set of TCGA COAD/READ. "WNT" depicts the CRC that carries oncogenic mutations in WNT pathway genes. "RAS" depicts CRC samples that have oncogenic mutations in RTK-RAS pathway genes. "P53" depicts CRC samples that have oncogenic mutations in TP53 pathway genes. All three pathways refer to the definition in The Cancer Genome Atlas Network paper (Nature 487, 330–337 (2012)).

Supplementary Figure S7 Kendall rank correlation test between MB and BMI (shaded area shows 95% confidence interval)

Supplementary Figure S8 Mutational burden in different bowel neoplasia. Mann-Whitney U test comparison shows no significance between large and small polyps in the groups of tubular adenomas (TA), tubulovillous adenoma (TVA) and sessile serrated lesions (SSL).

Supplementary Figure S9 Kendall rank correlation test between MB and polyp size (shaded area shows 95% confidence interval) in different types of polyps.

Supplementary Figure S10 Kendall rank correlation test between polyp burden and mutation burden. Statistic test shows a positive correlation between polyp burden and mutation burden in concurrent group but not in non-concurrent groups (shaded area shows 95% confidence interval).

Supplementary Figure S11 Immune cell deconvolution analysis on CD45+ segments.

Supplementary Table S1 Clinical characteristics of specimens involved in WES analysis

Supplementary Table S2 Formalin-fixed, paraffin-embedded (FFPE) polyp samples involved in GeoMx analysis

Supplementary Table S3 Somatic mutations in the whole sample cohort involved in WES

Supplementary Table S4 Somatic mutations in LGD polyps which carry concurrent oncogenic mutations in WNT/RTK-RAS/TP53 signalling pathways

Supplementary Table S5 Somatic mutations in LGD polyps with non-concurrent oncogenic mutations in WNT/RTK-RAS/TP53 signalling pathways

Supplementary Table S6 Oncogenic mutations in a 3 mm TA with MB of 6.45

Supplementary Table S7 Significantly differetial expressed genes between concurrent polyp epithelial and non-concurrent polyp epithelial segments

Supplementary Table S8 Statistical test - T.test on normalised enrichment scores, with BH-adjusted p-values
