# Supplementary figures and images for: Mutational Analysis of Early, Low-Grade Bowel Polyps Defines a Subgroup with Concurrent, High-Risk Oncogenic Drivers Independent of Polyp Size
Source: Cancer Res Commun. 2025 Aug 19;5(8):1372–83. doi: 10.1158/2767-9764.CRC-25-0182 (PMC12361885; doi:10.1158/2767-9764.CRC-25-0182)

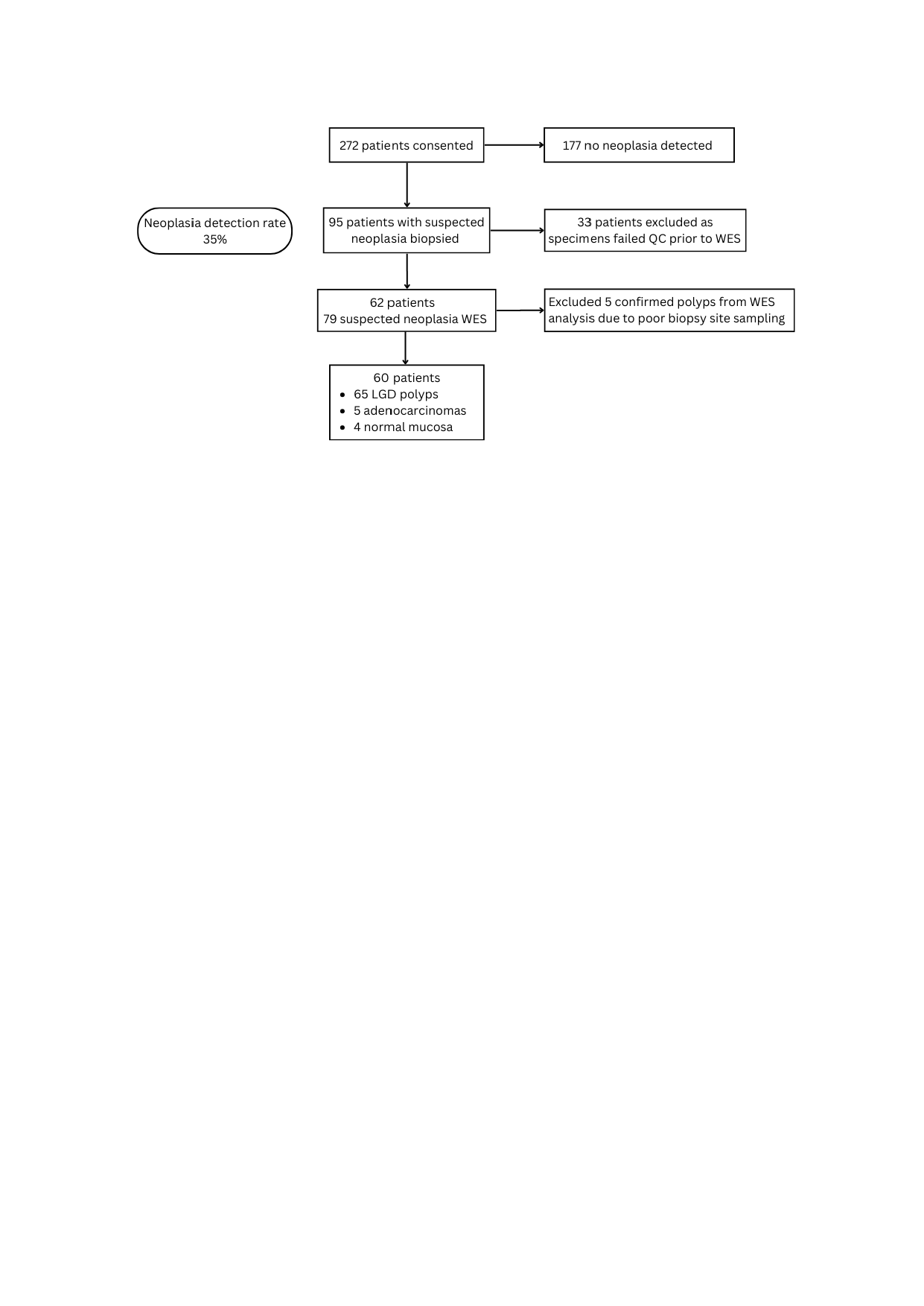

Supplement: Supplementary Figure S1 — Study recruitment and analysis workflow. [file crc-25-0182_supplementary_figure_s1_suppsf1.png]

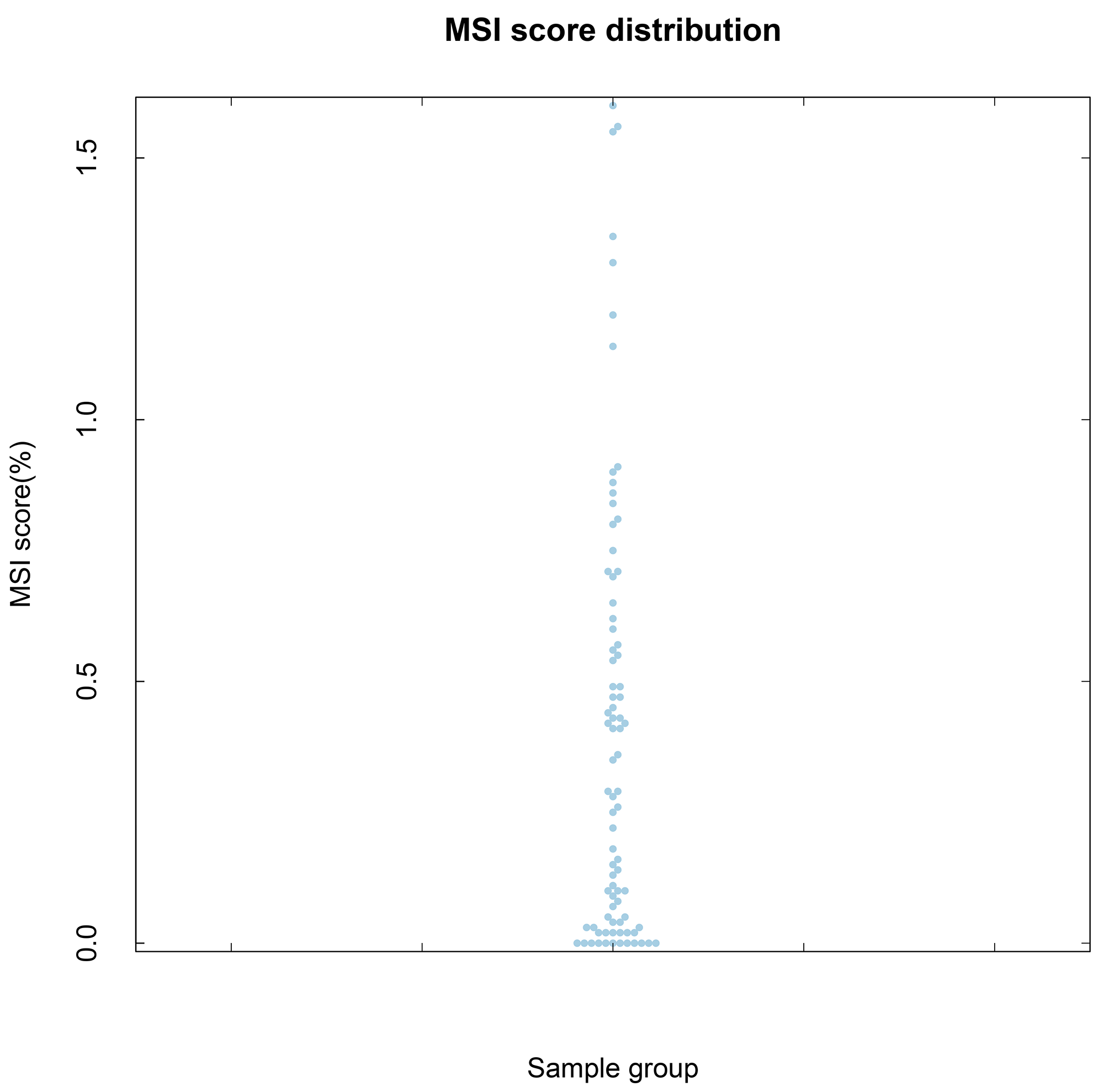

Supplement: Supplementary Figure S2 — MSI scores in the sample cohort, estimated by MSIsensor-pro. [file crc-25-0182_supplementary_figure_s2_suppsf2.png]

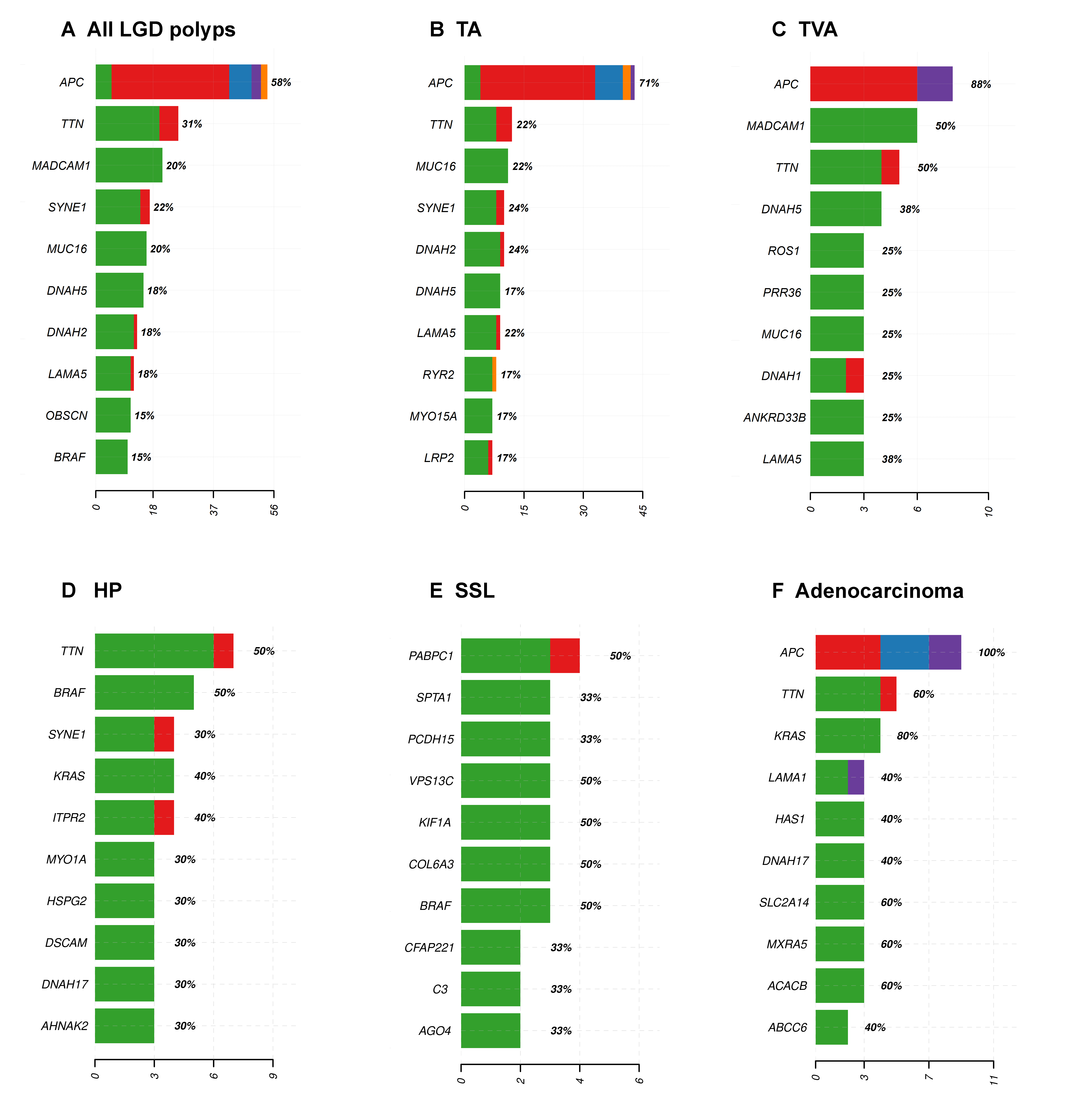

Supplement: Supplementary Figure S3 — Top ten mutated genes in each bowel neoplasia type. [file crc-25-0182_supplementary_figure_s3_suppsf3.png]

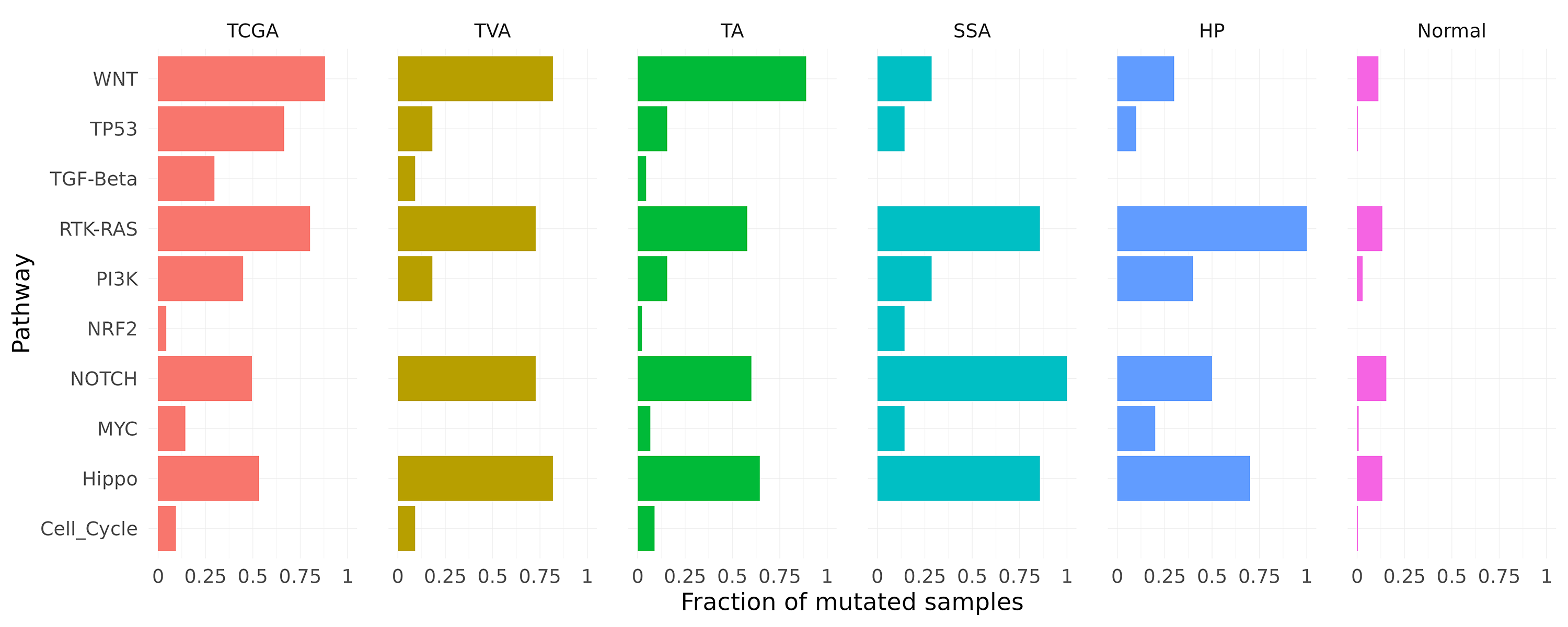

Supplement: Supplementary Figure S5 — The most frequently altered oncogenic pathways reported in TCGA adenocarcinoma data was mapped to bowel polyp specimens analyzed in this study (tubulovillous adenoma (TVA), tubular adenomas (TA), sessile serrated lesions (SSL) and hyperplastic (HP), and normal healthy colon data. [file crc-25-0182_supplementary_figure_s5_suppsf5.png]

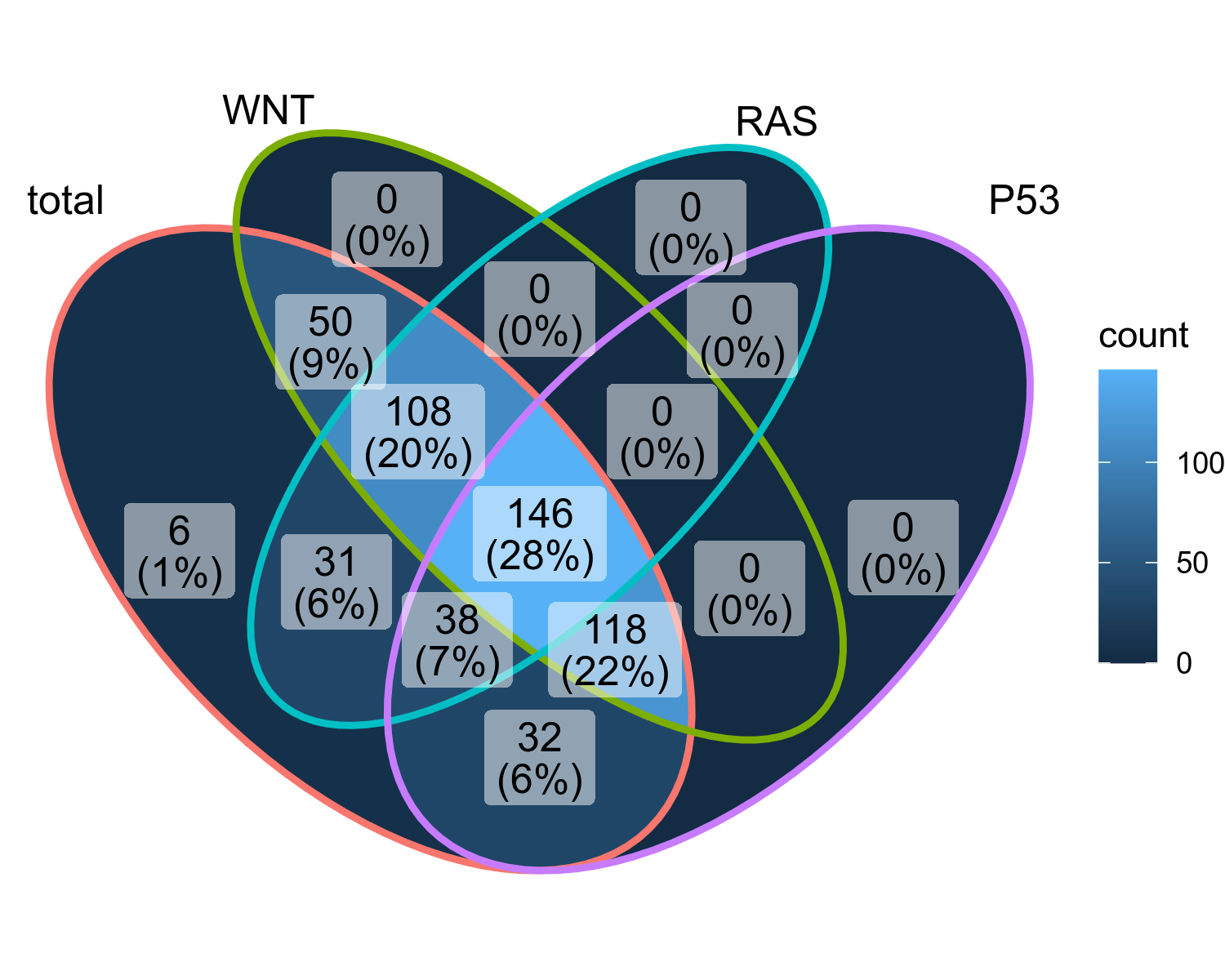

Supplement: Supplementary Figure S6 — Venn diagram to show the intersection between CRC carrying mutations in WNT, RAS and TP53 pathway from TCGA adenocarcinoma dataset. "total" indicates the whole sample set of TCGA COAD/READ. "WNT" depicts the CRC that carries oncogenic mutations in WNT pathway genes. "RAS" depicts CRC samples that have oncogenic mutations in RTK-RAS pathway genes. "P53" depicts CRC samples that have oncogenic mutations in TP53 pathway genes. All three pathways refer to the definition in The Cancer Genome Atlas Network paper (Nature 487, 330–337 (2012)). [file crc-25-0182_supplementary_figure_s6_suppsf6.png]

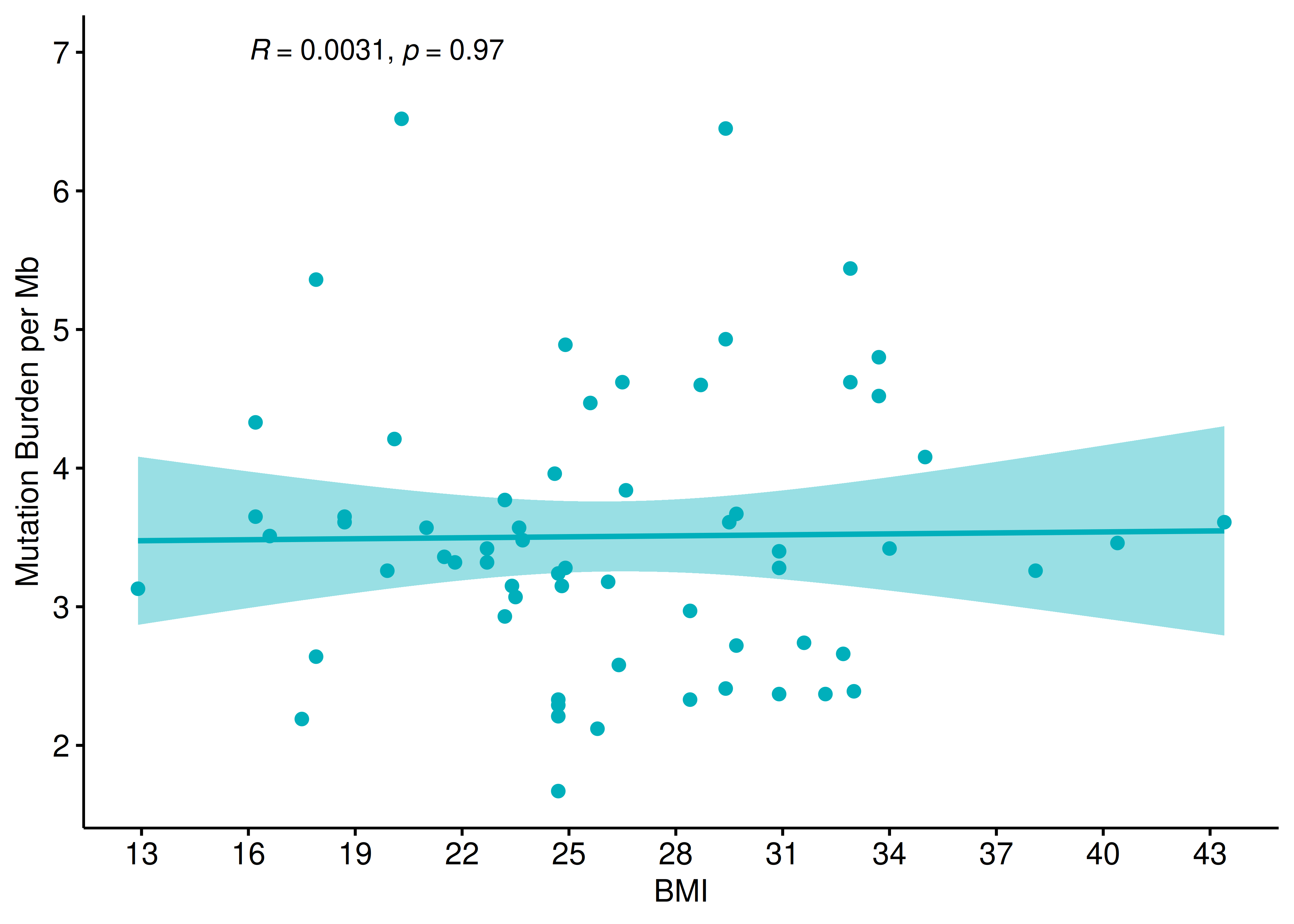

Supplement: Supplementary Figure S7 — Kendall rank correlation test between MB and BMI (shaded area shows 95% confidence interval) [file crc-25-0182_supplementary_figure_s7_suppsf7.png]

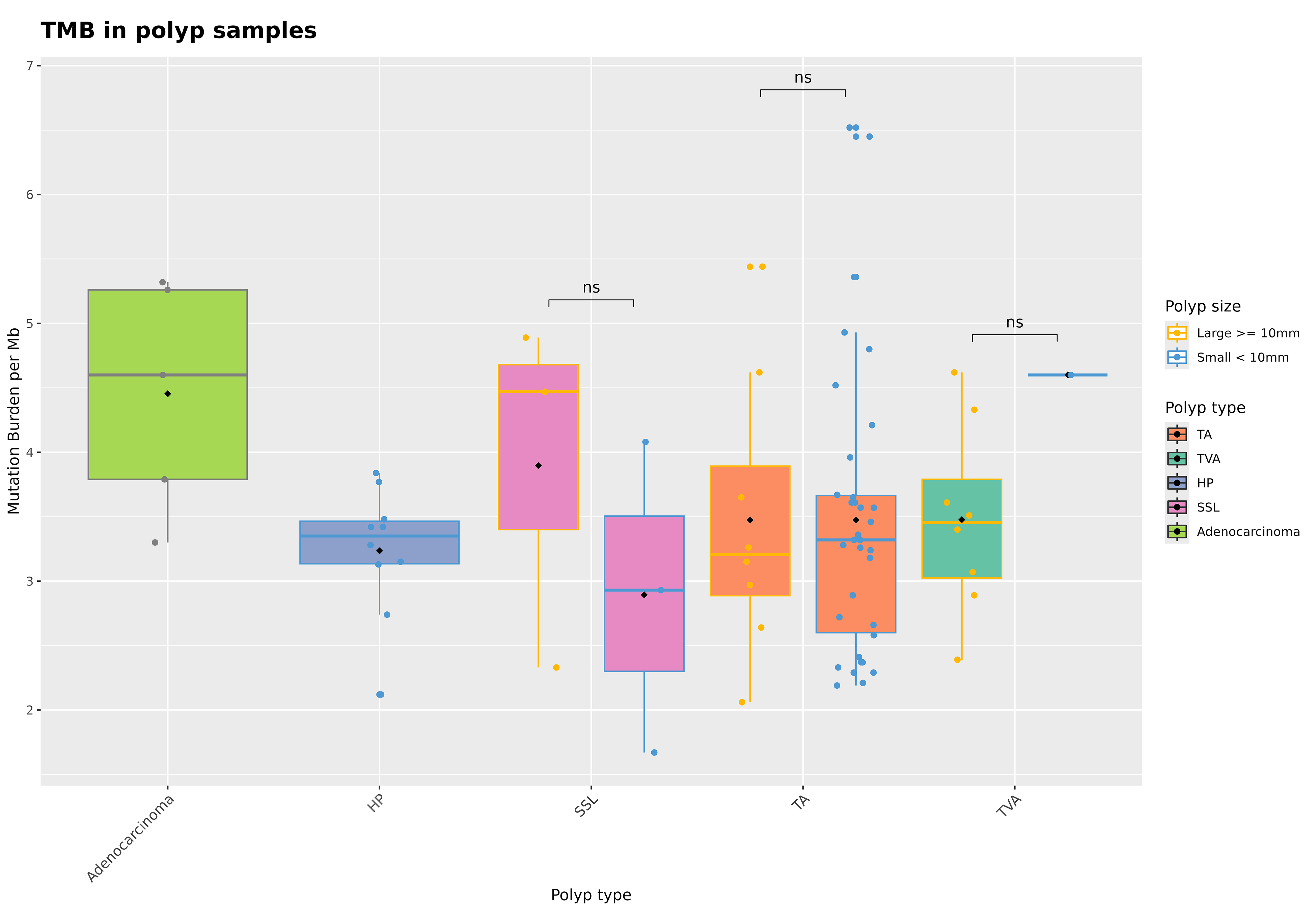

Supplement: Supplementary Figure S8 — Mutational burden in different bowel neoplasia. Mann-Whitney U test comparison shows no significance between large and small polyps in the groups of tubular adenomas (TA), tubulovillous adenoma (TVA) and sessile serrated lesions (SSL). [file crc-25-0182_supplementary_figure_s8_suppsf8.png]

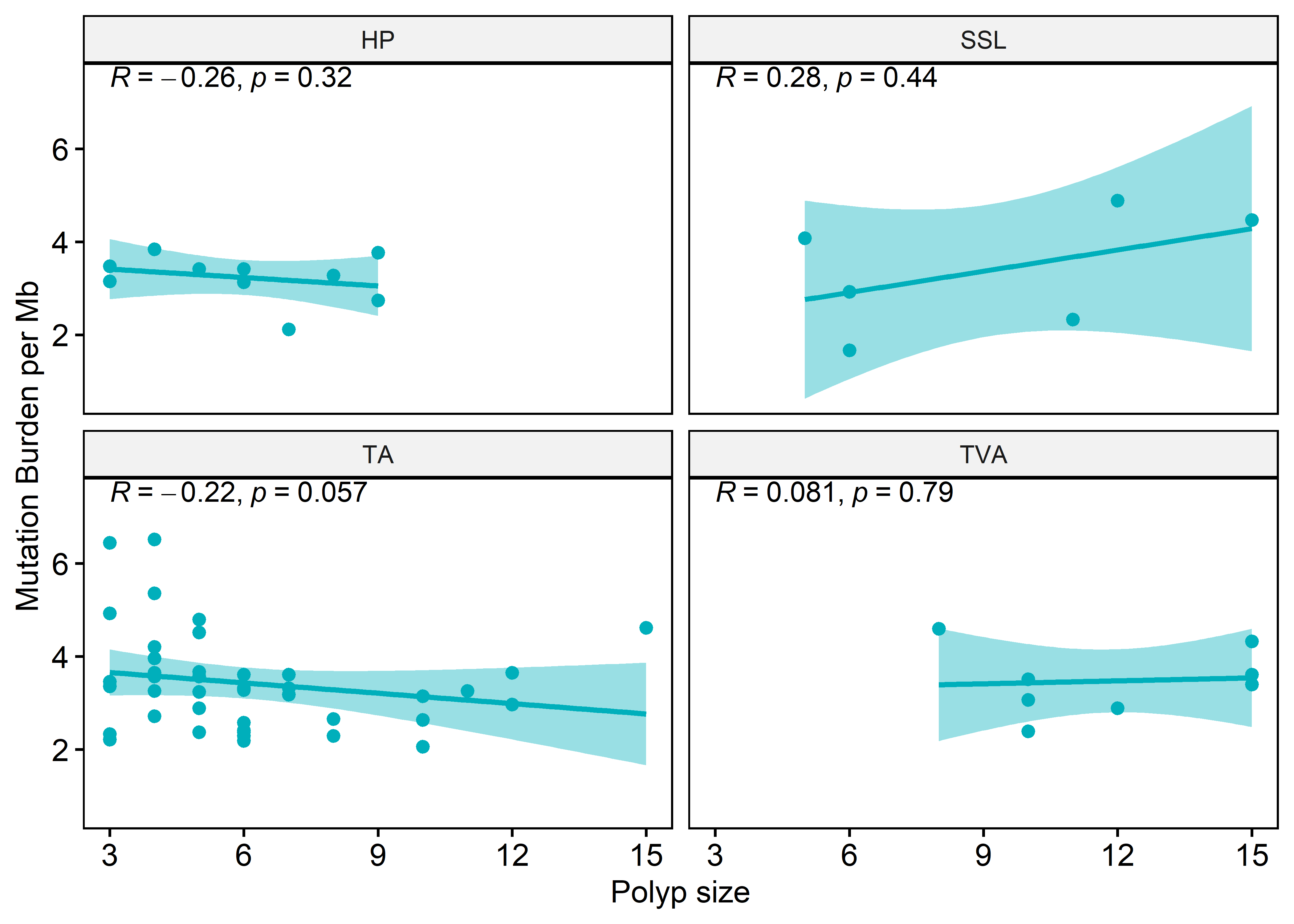

Supplement: Supplementary Figure S9 — Kendall rank correlation test between MB and polyp size (shaded area shows 95% confidence interval) in different types of polyps. [file crc-25-0182_supplementary_figure_s9_suppsf9.png]

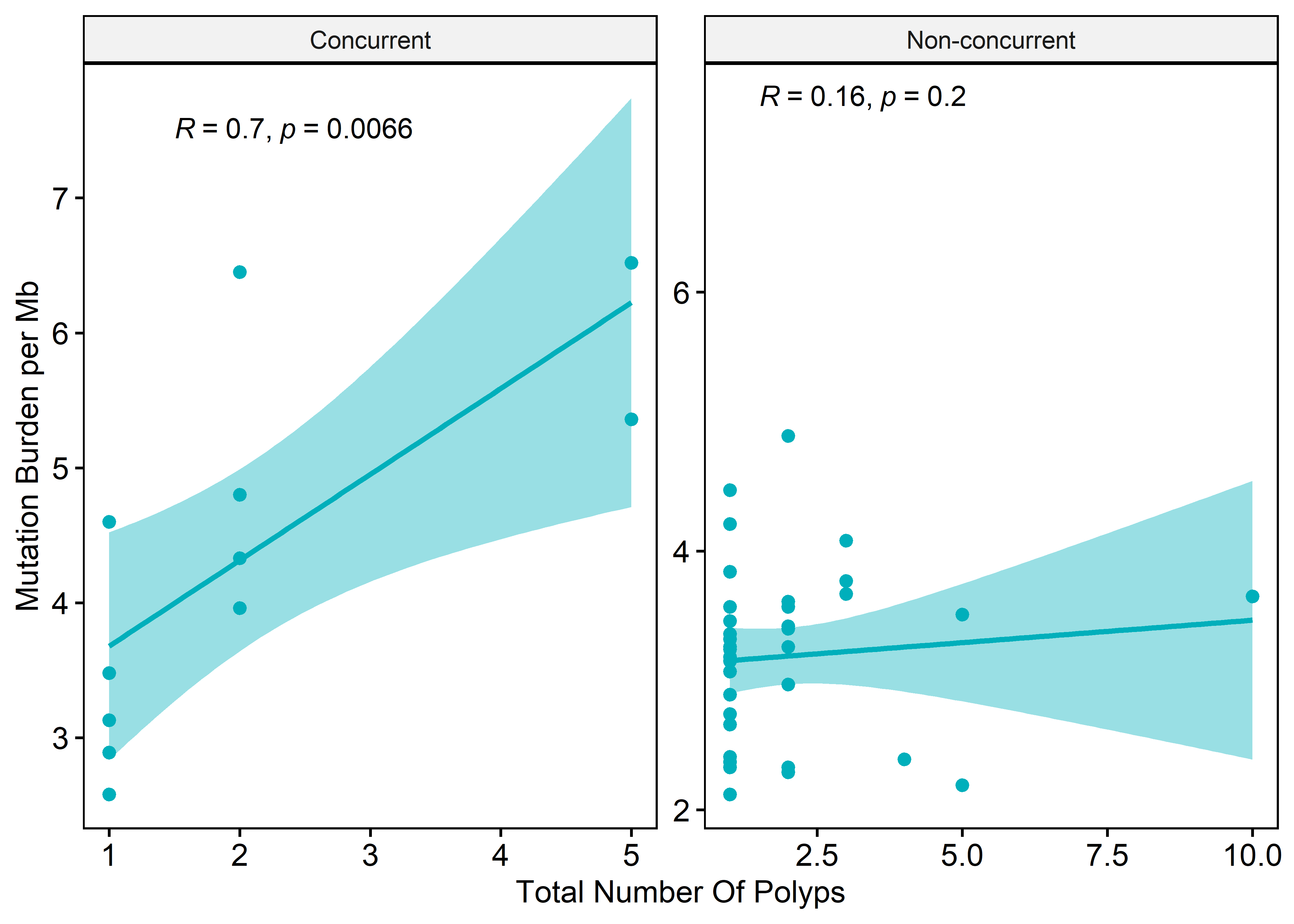

Supplement: Supplementary Figure S10 — Kendall rank correlation test between polyp burden and mutation burden. Statistic test shows a positive correlation between polyp burden and mutation burden in concurrent group but not in non-concurrent groups (shaded area shows 95% confidence interval). [file crc-25-0182_supplementary_figure_s10_suppsf10.png]

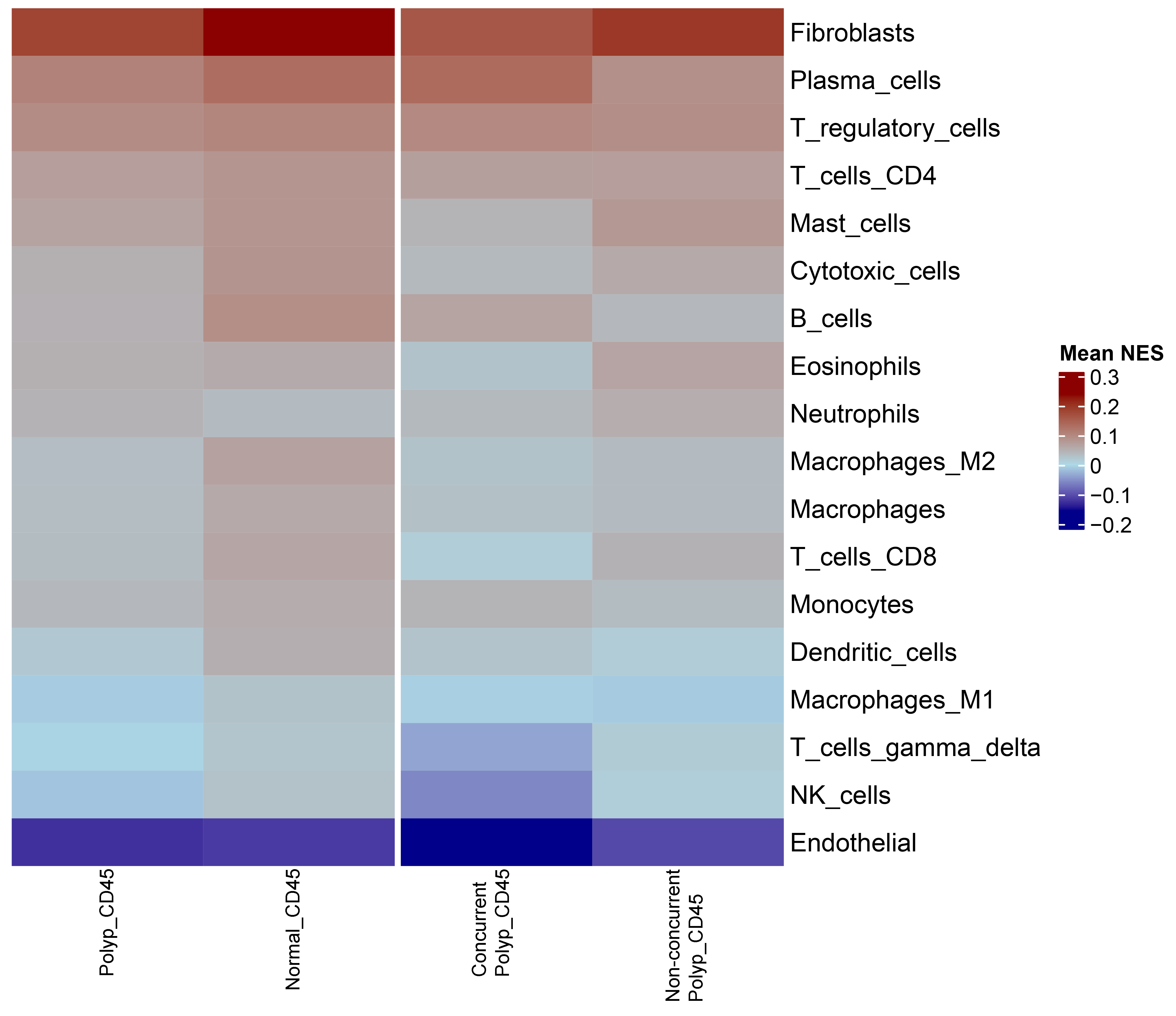

Supplement: Supplementary Figure S11 — Immune cell deconvolution analysis on CD45+ segments. [file crc-25-0182_supplementary_figure_s11_suppsf11.png]
